# The Effect of a Mediterranean Diet on the Incidence of Cataract Surgery

**DOI:** 10.3390/nu9050453

**Published:** 2017-05-03

**Authors:** Alfredo García-Layana, Gianfranco Ciufo, Estefania Toledo, Miguel A. Martínez-González, Dolores Corella, Montse Fitó, Ramon Estruch, Enrique Gómez-Gracia, Miguel Fiol, José Lapetra, Lluís Serra-Majem, Xavier Pintó, Maria P. Portillo, José V. Sorli, Mónica Bulló, Ernest Vinyoles, Aleix Sala-Vila, Emilio Ros, Jordi Salas-Salvadó, Fernando Arós

**Affiliations:** 1 Department of Ophthalmology, University of Navarra, 31008 Pamplona, Spain; gciufo@unav.es; 2 CIBER de la Fisiopatología de la Obesidad y la Nutrición (CIBEROBN), Instituto de Salud Carlos III, 28029 Madrid, Spain; etoledo@unav.es (E.T.); mamartinez@unav.es (M.A.M.-G.); dolores.corella@uv.es (D.C.); mfito@imim.es (M.F.); restruch@clinic.ub.es (R.E.); miguel.fiol@ssib.es (M.F.); jlapetra@ono.com (J.L.); lluis.serra@ulpgc.es (L.S.-M.); xpinto@bellvitgehospital.cat (X.P.); mariapuy.portillo@ehu.es (M.P.P.); sorli@uv.es (J.V.S.); monica.bullo@urv.cat (M.B.); asala@clinic.cat(A.S.-V.); EROS@clinic.ub.es (E.R.); jordi.salas@urv.cat (J.S.-S.); aborau@secardiologia.es (F.A.); 3 The PREDIMED (Prevención con Dieta Mediterránea) Research Network (RD/06/0045), Instituto de Salud Carlos III (ISC III), 28029 Madrid, Spain; egomezgracia@uma.es; 4 Department of Preventive Medicine and Public Health, University of Navarra, 31008 Pamplona, Spain; 5 Department of Preventive Medicine, University of Valencia, 46010 Valencia, Spain; 6 Cardiovascular Risk and Nutrition (Regicor Study Group), Hospital del Mar Medical Research Institute (IMIM), 08003 Barcelona, Spain; 7 Department of Internal Medicine, August Pi i Sunyer Institute of Biomedical Research (IDIBAPS), Hospital Clinic, University of Barcelona, 08036 Barcelona, Spain; 8 Department of Preventive Medicine, University of Malaga, 29016 Malaga, Spain; 9 Institute of Health Sciences, University of Balearic Islands and Son Espases Hospital, 07122 Palma de Mallorca, Spain; 10 Department of Family Medicine, Distrito Sanitario Atención Primaria Sevilla, Centro de Salud San Pablo, 41007 Sevilla, Spain; 11 Department of Clinical Sciences, University of Las Palmas de Gran Canaria, 35001 Las Palmas de Gran Canaria, Spain; 12 Lipids and Vascular Risk Units. Internal Medicine, University Hospital of Bellvitge, Hospitalet de Llobregat, 08907 Barcelona, Spain; 13 Nutrition and Obesity Group Department of Nutrition and Food Science, University of Basque Country and Lucio Lascaray Research Center, 48940 Vitoria, Spain; 14 Human Nutrition Unit, Faculty of Medicine and Health Sciences, IISPV, Rovira i Virgili University, 43003 Reus, Spain; 15 Cap La Mina, University of Barcelona, 08930 Barcelona, Spain; evinyoles@fundaciojgol.org; 16 Lipid Clinic, Endocrinology and Nutrition Service, IDIBAPS, Hospital Clinic, University of Barcelona, 08036 Barcelona, Spain; 17 Department of Cardiology, University Hospital Araba, 01009 Vitoria, Spain

**Keywords:** Mediterranean diet, PREDIMED, cataract, cataract surgery, nuts, extra-virgin olive oil, low-fat diet, antioxidants

## Abstract

Background: Cataract is a leading cause of vision impairment worldwide, and surgery is the only available treatment. The process that initiates lens opacification is dependent on the oxidative stress experienced by the lens components. A healthy overall dietary pattern, with the potential to reduce oxidative stress, has been suggested as a means to decrease the risk of developing cataract. We aimed to investigate the hypothesis that an intervention with a Mediterranean diet (MedDiet) rather than a low-fat diet could decrease the incidence of cataract surgery in elderly subjects. Methods: We included 5802 men and women (age range: 55–80 years) from the Prevención con Dieta Mediterránea study (multicenter, parallel-group, randomized controlled clinical trial) who had not undergone cataract surgery. They were randomly assigned to one of three intervention groups: (1) a MedDiet enriched with extra-virgin olive oil (EVOO) (*n* = 1998); (2) a MedDiet enriched with nuts (*n* = 1914), and a control group recommended to follow a low-fat diet (*n* = 1890). The incidence of cataract surgery was recorded yearly during follow-up clinical evaluations. Primary analyses were performed on an intention-to-treat basis. Cox regression analyses were used to assess the relationship between the nutritional intervention and the incidence of cataract surgery. Results: During a follow-up period of 7.0 years (mean follow-up period: 5.7 years; median: 5.9 years), 559 subjects underwent cataract surgery. Two hundred and six participants from the MedDiet + EVOO group, 174 from the MedDiet + Nuts group, and 179 from the control group underwent cataract surgery. We did not observe a reduction in the incidence of cataract surgery in the MedDiet groups compared to the control group. The multivariable adjusted hazard ratios were 1.03 (95% confidence interval [CI]: 0.84–1.26, *p* = 0.79) for the control group versus the MedDiet + EVOO group and 1.06 (95% CI: 0.86–1.31, *p* = 0.58) for the control group versus the MedDiet + Nuts group. Conclusions: To our knowledge, this is the first large randomized trial assessing the role of a MedDiet on the incidence of cataract surgery. Our results showed that the incidence of cataract surgery was similar in the MedDiet with EVOO, MedDiet with nuts, and low-fat diet groups. Further studies are necessary to investigate whether a MedDiet could have a preventive role in cataract surgery.

## 1. Introduction

The prevalence of cataract is high among elderly individuals, and it is the primary cause of blindness worldwide [1]. The number of people with age-related cataract has been predicted to increase dramatically in the next 20 years worldwide, especially in Western countries, because of the increasing life expectancy [2]. The only available treatment to restore vision after cataract formation is via intraocular surgery, and this imposes a major medical cost and heavy workload burden on the health care systems [3].

Oxidative stress is believed to be involved in the formation and maturation of cataract, causing damage to the proteins and lipids of the lens epithelium [4,5,6]. Age, ultraviolet light exposure, smoking, and corticosteroid use are recognized risk factors associated with cataract formation [7,8]. Besides other risk factors such as abdominal obesity [9] and hormonal therapy [10], the production of proinflammatory components increases the levels of free oxygen radicals [11,12], which have been associated with an increasing risk of cataract.

A significant and growing body of observational research suggests that an appropriate nutritional intervention may offer a way to decrease the risk of cataract [13]. Accordingly, the association between cataract progression and nutritional exposure is a matter of great scientific interest. Recently, an inverse association between the total antioxidant capacity (TAC) and the risk of age-related cataract has been reported [8]. In some trials, free oxygen radicals have been identified as one of the most important causes of cataract formation [10]. A fair number of cohort studies have focused on assessing the association between the intake of several specific nutrients in the diet and cataract formation [11,12,13,14,15,16,17,18,19]. In some of those studies, an antioxidant-rich diet was reported to potentially delay cataract progression [20]; other studies have reported that healthier diets could prevent cataract formation [21,22,23,24,25]. Some nutritional supplements, including different antioxidants [26], lutein/zeaxanthin [27,28,29,30] and omega-3 [31] and omega-6 [32] fatty acids, have been suggested to reduce cataract progression.

The Mediterranean diet (MedDiet) is recognized as one of the healthiest dietary patterns and has proven beneficial for several health outcomes [33,34]. However, no randomized trial to date has assessed the long-term effect of a MedDiet on the risk of cataract development in a large population of elderly subjects. We hypothesized that two MedDiets, one enriched with extra-virgin olive oil (EVOO) and another with mixed nuts, rather than a low-fat control diet could decrease the incidence of cataract surgery in a population at a high risk of cardiovascular disease (CVD) enrolled to the Prevención con Dieta Mediterránea (PREDIMED) trial.

## 2. Methods

### 2.1. PREDIMED Trial

The analysis was conducted within the framework of the PREDIMED study (www.predimed.es) [35], a parallel-group, randomized, primary CVD prevention trial in persons at high risk of CVD. The main results of the trial related to the primary cardiovascular endpoint have been published elsewhere [36]. The study was conducted in accordance with the Declaration of Helsinki. The Institutional Review Board of the respective recruitment centers approved the study protocol and all participants gave their informed consent. Trial Registration: clinicaltrials.gov Identifier: ISRCTN35739639.

### 2.2. Participants

Participants were men (age range: 55–80 years) or women (age range: 60–80 years) initially free of CVD. The inclusion criteria were the presence of either type 2 diabetes or ≥3 major cardiovascular risk factors, including current smoking (>1 cigarette/day during the last month), hypertension (systolic BP ≥140 mmHg or diastolic BP ≥90 mmHg or under antihypertensive medication), LDL cholesterol ≥160 mg/dL or receiving lipid-lowering therapy, HDL cholesterol ≤40 mg/dL in men or ≤50 mg/dL in women, body mass index (BMI) ≥25 kg/m^2^, and a family history of premature coronary heart disease [35].

The exclusion criteria were a previous history of CVD (i.e., a previous medical diagnosis of acute myocardial infarction, stroke, or peripheral arterial disease), any severe chronic illness, immunodeficiency or human immunodeficiency virus positivity, illegal drug or alcohol abuse, history of allergy to olive oil or nuts, and low predicted likelihood of changing dietary habits according to the Prochaska and DiClemente stages of change model [35].

In total, 7447 participants were enrolled in the PREDIMED study. The selection process started by extracting the names of potential participants from the records of around 200 primary care centers (PCCs) affiliated with 11 Spanish teaching hospitals between October 2003 and January 2009. The clinical records of these participants were then individually reviewed to exclude those who did not meet the eligibility criteria. Potential participants were approached by the PCCs via a telephone call or during their clinical visits. If the candidates were interested in participating, a face-to-face interview was scheduled. During this interview, the purpose and characteristics of the study were explained, and signed informed consent was obtained from willing participants. A brief explanation of the study, including the possibility that they might receive free allowances of EVOO or nuts for the duration of the trial, was given at this first visit [35].

### 2.3. Randomization and Intervention

Once entered into the study, the participants were randomly assigned in a 1:1:1 ratio to one of the following three intervention groups: (1) MedDiet supplemented with EVOO (MedDiet + EVOO); (2) MedDiet supplemented with mixed nuts (MedDiet + Nuts); or (3) a control diet (a low-fat diet according to the American Heart Association guidelines applicable as of 2002). The two groups allocated the MedDiets received intensive education to follow the MedDiet and supplemental foods at no cost. EVOO (1 L/week for the participant and his/her family) was provided to the MedDiet + EVOO group, and mixed nuts (30 g/day; 15 g walnuts, 7.5 g hazelnuts, and 7.5 g almonds) were provided to the MedDiet + Nuts group. The participants in the control group did not receive education on the MedDiet, but were adviced to follow a low-fat diet [35].

Randomization was performed centrally by means of a computer-generated random-number sequence. Investigators and members of all committees were blinded to the treatments assigned to individual participants. In the present analysis, our main objective was to determine the effect of the three dietary interventions on the incidence of cataract surgery.

### 2.4. Follow-Up and Adherence

At baseline and during the yearly follow-up visits, all participants underwent clinical evaluations with their general practitioners. In addition they completed a 14-item questionnaire to assess adherence to the MedDiet; a 77-item general questionnaire about lifestyle variables, educational achievement, history of illnesses (including cataract surgery), and medication use; a 137-item validated food-frequency questionnaire [36]; and a validated Spanish version of the Minnesota Leisure-Time Physical Activity Questionnaire.

Biomarkers of adherence to the MedDiet interventions were measured in a random sample of PREDIMED participants during the first 5 years of follow-up, and included urine hydroxytyrosol concentrations and plasma α-linolenic acid proportions, which are reliable biomarkers of EVOO and walnut intake, respectively [36]. General practicioners and laboratory technicians were blinded to the participants’ intervention groups.

### 2.5. Outcome Measures

The outcome we investigated was the occurrence of cataract surgery at any time throughout the study period. Cataract surgery (externally confirmed by an independent Adjudication Committee blinded to the intervention and to the dietary habits of the participants) was a prespecified secondary outcome of the PREDIMED trial. Patients with cataract surgery in any eye present at baseline were excluded from the analyses. For the assessment of incident cataract surgery during the follow-up period, the participants visited their general practitioners yearly for clinical evaluations and completed several questionnaires, including a general medical questionnaire recording changes in the health status and, specifically, if they had undergone cataract surgery. The cataract surgery outcome was defined by the medical diagnosis made by an ophthalmologist, and was explicitly reported in the medical charts. The occurrence of cataract surgery was also confirmed by periodically reviewing the computer-based records of the corresponding PCCs. These reports and all relevant documentation, including medical records made by the ophthalmologist, were sent to the PREDIMED members of the Clinical Adjudication Event Committee, who were blinded to the interventions. As cataract extraction was a predefined secondary endpoint in the trial, the Adjudication Committee reviewed the medical charts comprehensively for potential cataract extraction, and only definitively confirmed cases were included in this analysis. Cases of traumatic cataracts and cases that appeared after another intraocular surgery, such as vitrectomy or glaucoma surgery, were also excluded. In cases of bilateral surgery in the same patient, only the first event was considered in our time-to-event analyses. Our analyses focused on a subset of 5802 participants from the PREDIMED trial who had not undergone cataract surgery at baseline: 1998, 1914, and 1890 participants were allocated to the MedDiet + EVOO, MedDiet + Nuts, and control diet groups, respectively. The remaining 1645 participants enrolled in the PREDIMED study were excluded because they had undergone cataract surgery before the beginning of the trial.

### 2.6. Statistical Analysis

Baseline differences between the three dietary intervention groups were tested using analysis of variance or chi-squared tests, and results were expressed as means ± SD or numbers (percentages), respectively. The normality of variables was examined by using the Kolmogorov-Smirnov test. All analyses were performed on the basis of an intention-to-treat principle. 

Person-time of follow-up was calculated as the interval between the randomization date and the earliest date of the follow-up contact at which a new cataract surgery was recorded, or the last visit or death, whichever came first.

We used unadjusted, age- and sex-adjusted, and multivariable time-dependent Cox proportional hazard models to assess the effect of the two MedDiet interventions on the incidence of cataract surgery in comparison with the control group. Hazard ratios (HRs) and 95% confidence intervals (CIs) were calculated using the control group as the reference. In multivariable models, we adjusted the estimates for the following confounders: age, sex, baseline type 2 diabetes, baseline hypertension, baseline BMI (4 categories), and smoking status (3 categories). All models were stratified by recruitment center. Prespecified interactions with sex, age, or baseline type 2 diabetes were tested using the likelihood ratio test in fully adjusted models. A fully adjusted multivariable analysis was repeated after both the MedDiet groups were merged into a single category for comparison with the control group. The assumption of proportional hazards was tested by analyzing the scaled Schoenfeld residuals, and it was not violated (*p* > 0.50). The test for time-varying covariates also suggested that the assumption of proportional hazards was met. We also used the Kaplan-Meier method to graphically estimate the cumulative incidence of cataract surgeries. A 2-tailed *p* value < 0.05 was considered statistically significant. In addition, we performed prespecified subgroup analysis within the strata of age, sex, and BMI. Analyses were performed using IBM SPSS Statistics for Windows Version 19.0 (IBM Corp., Armonk, NY, USA) and Stata 12.0 (StataCorp, College Station, TX, USA).

## 3. Results

The overall baseline characteristics and intake of energy and nutrients as well as the consumption of key foods by the study participants in the dietary intervention groups are listed in Table 1 and Table 2, respectively. The mean age of the participants was 66 years and their mean BMI was 30.1 kg/m^2^; 45% of them were men. The participants in all three intervention groups were well matched for age, sex, anthropometric features, and other cardiovascular risk factors (data not shown).

During the follow-up period, 559 of the 5802 participants (9.6%) from the three intervention groups underwent cataract surgery, including 206 (10.3%) from the MedDiet + EVOO group, 174 (9.1%) from the MedDiet + Nuts group, and 179 (9.4%) from the control group. It must be noted that the number of participants who underwent cataract surgery does not correspond with the total number of eyes that underwent surgeries because we considered only the number of participants operated, regardless of whether they underwent surgery in one or both eyes.

**Figure 1:**
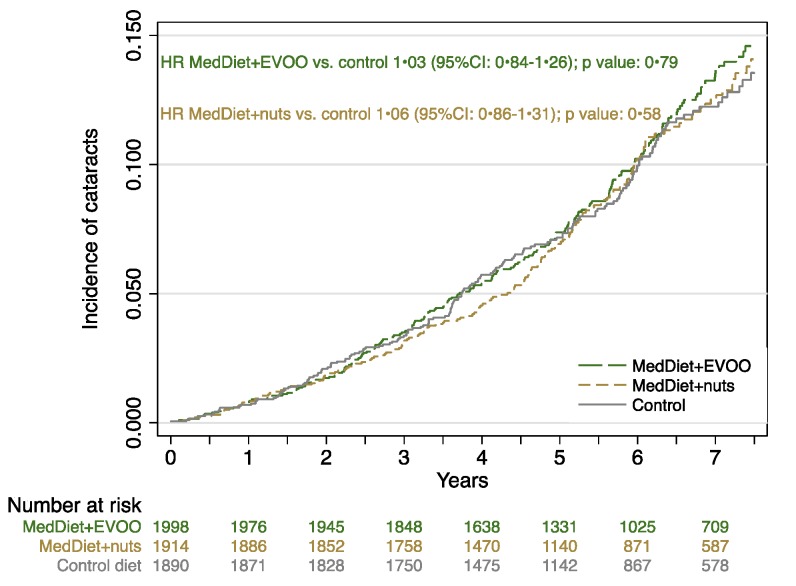
Incidence of cataract surgery during follow-up among the 3 interventions group.

We did not observe a reduction in the incidence of cataract surgery in the groups assigned to the MedDiet versus the control group. The observed rates (per 1000 person-years) were 16.9, 17.6, and 16.2 for the MedDiet + EVOO, MedDiet + Nuts, and control groups, respectively (Table 3). Figure 1 displays the incidence of cataract surgery and the HRs and their 95% CIs for the effect of the two MedDiet interventions in comparison with the control group. Compared with the control group, the MedDiet + EVOO group had an unadjusted HR for the incidence of cataract surgery of 1.02 (95% CI, 0.84–1.25) and the MedDiet + Nuts group had a value of 0.97 (95% CI, 0.78–1.19). When we adjusted for some possible confounders (age, sex, baseline type 2 diabetes, baseline hypertension, baseline BMI, and smoking status), we did not observe a significant difference between the MedDiet groups and the control group, with multivariable-adjusted HR of 1.03 (95% CI, 0.84–1.26) for the MedDiet + EVOO group and 1.06 (95% CI, 0.86–1.31) for the MedDiet + Nuts group. Moreover, no significant difference was observed in the incidence of cataract surgery between the control group and both the MedDiet groups, when combined. In addition, we did not find any meaningful difference between the control group and the MedDiet groups when we analyzed for different subgroups of age, sex, and BMI (Table 4).

## 4. Discussion

Our analysis in the setting of the PREDIMED trial, including a middle-aged and elderly population with three or more CVD risk factors, was not able to confirm the initial hypothesis, because after 7.0 years of follow-up, we did not observe any significant differences in the incidence of cataract surgery between the participants assigned to the two MedDiet groups and the control group. The results showed that for both the MedDiet groups, the HRs for the incidence of cataract surgery were near the reference (null) value of 1.0. The results also remained non-significant after adjusting for certain potential confounders and analyzing the different subgroups according to age, sex, and BMI. Our findings are in accordance with those of good-quality trials, such as the Age-Related Eye Disease Study, and prospective epidemiological studies [37] that did not provide support for a beneficial effect of antioxidants in reducing the risk of cataract formation or extraction. However, we acknowledge that other studies did show a significant reduction in cataract incidence or progression associated with the intake of antioxidants [13,14,15,16,17,18,19].

The primary aim of the PREDIMED trial was to assess the role of a MedDiet in the primary prevention of cardiovascular events [36].The main focus of the intervention was to change the overall dietary pattern, adding to a healthy diet the extra benefit of monounsaturated and polyunsaturated fatty acids and other nutrients provided by EVOO and nut supplements, instead of focusing on changes in single macronutrients or micronutrients. In contrast with the null effect on cataract surgery, both the MedDiets supplemented with EVOO or nuts were beneficial in reducing the risk of major cardiovascular events [36], when compared to a low-fat diet, thereby demonstrating the protective effects of the MedDiet including EVOO and nuts.

To our knowledge, this study is the first large randomized trial to assess the effects of a MedDiet on the incidence of cataract. Numerous studies have investigated the association between diet and cataract and have focused on the effects of single micronutrients with antioxidant properties (e.g., vitamins A, B, C, and E; lutein/zeaxanthin; beta-carotene; or selenium). Although several of them have demonstrated an inverse relationship between these micronutrients and the development of age-related cataract or the incidence of cataract surgery, others have reported inconsistent results [38,39], showing no consensus about their importance. Instead, other studies have focused on the TAC [8], taking into account the capacity of all antioxidants in the diet and their synergic effect, demonstrating that dietary TAC was inversely associated with the risk of age-related cataract. Other trials have focused on the effects of single macronutrients such as lipids, and several have reported an inverse association between the intake of polyunsaturated fatty acids, like omega-3 and omega-6 fatty acids, and the risk of cataract [31]. The MedDiet is considered one of the healthiest dietary patterns and contains all the individual micronutrients that have an antioxidant effect. In our study, we tested the combined benefits of a MedDiet as well as EVOO and nuts that contain significant amounts of omega-3 fatty acids and bioactive compounds (including fiber, minerals, tocopherols, phytosterols, and phenolic compounds) with strong antioxidant effects. However, despite combining the effects of single antioxidants and the benefits provided by EVOO and nuts, the MedDiet used in our study did not show any superiority in reducing the incidence of cataract surgery when compared to a low-fat diet. Lu et al. [40] observed that saturated and polyunsaturated fats were not associated with the risk of cataract extraction, even though total fat intake was marginally associated with an increased risk of cataracts [31,40]. We cannot exclude the fact that the low-fat diet followed in our control group may have masked the potential benefit of the MedDiet.

The present study has some limitations and strengths that should be considered. The first limitation is the type of population participating in the study. As the participants were individuals with either type 2 diabetes or several CVD risk factors, like obesity and smoking, our findings cannot be extrapolated to younger subjects or to other populations. Moreover, these factors lead to the production of proinflammatory and oxidative agents and might play important roles in accelerating cataract formation. Furthermore, having type 2 diabetes could influence the strategy of when to operate a cataract, because in these patients, earlier cataract extraction has been known to contribute to an improved visual outcome [41]. Second, the timing of cataract extraction depends on multiple factors that can speed up or delay the surgery, including the ophthalmologist’s subjective decision; the patient’s visual acuity, comorbidities, and patient waiting attitude; being a more objective outcome formation of cataract diagnosed by slit lamp examination. Third, to assess the incidence of cataract surgery we considered only one surgery event in every patient, even though the same patient underwent cataract surgery in both eyes. This could have led to the underestimation of the real incidence of cataract surgery in our cohort, because other similar trials have observed an incidence of cataract surgery between 17.7% and 26.8% [42,43] , which was very different from our findings. Fourth, previous studies have shown that a high total dietary fat intake is related to a greater risk of developing cataracts [40], because dietary fat may affect lens cell membrane composition and function, which are related to age-related cataract [44]. Although there is lack of evidence, a low-fat diet could have a preventive role on the risk of cataract extraction. In our study, this diet could have masked the potential beneficial effect of the MedDiet on the incidence of cataract surgery, because a low-fat diet (control group) could be as effective as the MedDiet (intervention groups) in reducing the incidence of cataract surgery, without showing any difference between the two groups. Fifth, considering the outcome we investigated in this study, it could have been useful the additional presence of a fourth group (second control group) following no specific diet (neither MedDiet nor low-fat diet) to compare it with the three groups of the study to obtain information that could have indicated whether the MedDiet or the low-fat diet had any correlation with the incidence of cataract surgery, as comparing only the study groups we could not find any correlation. Sixth, the assessment of cataract surgery was not the primary endpoint, because the PREDIMED trial was designed to assess the effect of the MedDiet on primary CVD prevention. Lastly, our implicit assumption regarding the induction period for relating the diet to cataract surgery could be regarded as insufficient, even if the induction period was 6–7 years in our study. Given that cataract surgery implies there was a long-standing previous pathophysiological process in the lens, which probably started many years before our recorded date of surgery, our assumption regarding the induction period could provide an alternative explanation to our null results. We admit that we may have reduced our sensitivity in identifying the cataract-related endpoint. However, the advantage of using surgery in our operational definition of this endpoint is its high specificity. Moreover, it is well known in epidemiology that, theoretically, with perfect specificity, the nondifferential sensitivity of disease misclassification will not bias the relative risk estimate [45].

The main strengths of our study are its randomized design; long follow-up period relative to previous studies; large sample size; good compliance with the dietary regimens assigned, which is further supported by the MedDiet questionnaire results and changes in the biomarkers of food supplementation; and the control for several potential confounders, which together with the randomization allows us to rule out residual confounding.

## 5. Conclusions

In summary, the results of our analysis do not support that a MedDiet supplemented with EVOO or nuts may reduce the incidence of cataract surgery when compared to a low-fat diet in a population at a high risk of CVD. However, the present results should be interpreted with caution because the absence of evidence does not indicate the lack of evidence of an effect. Further studies on a healthy population, taking into account alternative definitions of the outcome, are needed to investigate the association between the MedDiet and age-related cataract and to determine whether the MedDiet could have a potential preventive role in cataract.

## Figures and Tables

**Table 1:** Baseline Characteristics of the PREDIMED Trial Participants by Intervention Group.

Characteristic	Mediterranean Diet with EVOO	Mediterranean Diet with Nuts	Control Diet
	*N* = 1998	*N* = 1914	*N* = 1890
Age—year (mean ± SD)	66.1 ± 6.1	65.8 ± 5.9	66.3 ± 6.2
Sex (female)—No. (%)	1120 (56.1)	985 (51.5)	1099 (58.2)
Smoking—No. (%)			
Never smoker	1189 (59.5)	1117 (58.4)	1147 (60.7)
Former smoker	510 (25.5)	497 (26.0)	449 (23.8)
Current smoker	299 (15.0)	300 (15.7)	294 (15.6)
Body mass index † (mean ± SD)	30.0 ± 3.7	30.0 ± 3.8	30.3 ± 4.1
Waist to height ratio (mean ± SD)	0.63 ± 0.06	0.62 ± 0.06	0.63 ± 0.07
Waist circumference (mean ± SD)	100 ± 10	100 ± 11	101 ± 11
Hypertension ‡—No. (%)	1634 (81.8)	1591 (83.1)	1577 (83.4)
Type-2 diabetes §—No. (%)	970 (48.6)	851 (44.5)	897 (47.5)
Dyslipidemia ¶—No. (%)	1440 (72.1)	1414 (73.9)	1369 (72.4)
Family history of premature CHD—No. (%) |	447 (22.4)	426 (22.3)	442 (23.4)
Physical activity—METS-min/day (mean ± SD)	232 ± 229	248 ± 249	213 ± 236

Plus–minus values are means ± SDs. † The body-mass index (BMI) is the weight in kilograms divided by the square of the height in meters. ‡ Hypertension was defined as systolic blood pressure ≥140 mm Hg, diastolic blood pressure ≥90 mm Hg, or use of antihypertensive therapy. § Diabetes was defined as fasting blood glucose ≥126 mg/dL (7.0 mmol/L) on two occasions, or 2-h plasma glucose ≥200 mg/dL (11.1 mmol/L) after a 75-g oral glucose load, or use of antidiabetic medication. ¶ Dyslipidemia was defined as low-density lipoprotein cholesterol >160 mg/dL, high-density lipoprotein cholesterol ≤40 mg/dL in men or ≤50 mg/dL in women, or use of lipid-lowering therapy. | A family history of premature coronary heart disease (CHD) was defined as diagnosis of the disease in a male first-degree relative before the age of 55 years or in a female first-degree relative before the age of 65 years.

**Table 2:** Baseline intake of Energy, Nutrients and Key Foods of the PREDIMED Trial Participants by Intervention Group.

Variable	Mediterranean Diet with EVOO	Mediterranean Diet with Nuts	Control Diet
	*N* = 1998	*N* = 1914	*N* = 1890
Total energy intake (kcal/day) (mean ± SD)	2288 ± 612	2329 ± 620	2222 ± 602
Carbohydrate (g/day) (mean ± SD)	238.8 ± 81.7	242.6 ± 83.4	234.3 ± 79.2
Fiber (g/day) (mean ± SD)	25.4 ± 9.1	25.9 ± 9.2	24.7 ± 8.6
Protein (g/day) (mean ± SD)	93.3 ± 24.3	94.4 ± 23.2	90.4 ± 22.3
Fat (g/day) (mean ± SD)	99.5 ± 30.5	101.3 ± 30.2	96.3 ± 31.0
Saturated fatty acids	25.4 ± 9.1	25.8 ± 9.1	24.8 ± 9.3
Monounsaturated fatty acids	49.6 ± 16.0	50.0 ± 15.6	47.7 ± 16.5
ω-6 Polyunsaturated fatty acids	13.0 ± 6.4	13.7 ± 6.6	12.8 ± 6.2
ω-3 Marine PUFA	1.39 ± 0.75	1.51 ± 0.79	1.32 ± 0.65
Cholesterol (mg/day) (mean ± SD)	369.1 ± 137.5	374.3 ± 130.4	362.1 ± 128.5
Cereals (g/day) (mean ± SD)	231.4 ± 114.1	234.6 ± 108.6	225.6 ± 106.0
Vegetables (g/day) (mean ± SD)	344.5 ± 159.6	339.4 ± 152.1	324.8 ± 143.9
Fruits (g/day) (mean ± SD)	369.4 ± 212.5	370.2 ± 203.1	357.1 ± 200.6
Total nuts (g/day) (mean ± SD)	9.6 ± 13.9	12.5 ± 15.2	8.9 ± 12.6
Dairy Products (g/day) (mean ± SD)	378.8 ± 214.0	374.0 ± 220.6	376.0 ± 226.0
Red Meat (g/day) (mean ± SD)	134.7 ± 63.2	137.4 ± 59.8	130.0 ± 55.8
Seafood (g/day) (mean ± SD)	103.5 ± 54.7	102.4 ± 53.4	98.4 ± 48.2
Olive Oil (g/day) (mean ± SD)	40.5 ± 17.9	39.8 ± 17.4	38.4 ± 18.6
Alcohol consumption (g/day) (mean	9.1 ± 14.9	9.8 ± 15.5	7.9 ± 13.5
MedDiet Adherence score ‖ (mean ± SD)	8.7 ± 2.0	8.8 ± 2.0	8.4 ± 2.1

Mediterranean diet (MedDiet) adherence score (minimum adherence = 0 points; maximum adherence = 14 points). EVOO denotes extra virgin olive oil.

**Table 3:** Hazard ratios (95% CI) for the risk of cataract, according to the intervention group.

	Control Diet	Mediterranean Diet with EVOO	Mediterranean Diet with Nuts
	(*n* = 1890)	(*n* = 1998)	(*n* = 1914)
Cases/Person-years	179/10,633	206/11,728	174/10,719
Rate (/1000 person-years)	16.9	17.6	16.2
Crude rate ratio	1 (ref)	1.02 (0.84 to 1.25)	0.97 (0.78 to 1.19)
Multivariable adjusted rate ratio *	1 (ref)	1.03 (0.84 to 1.26)	1.06 (0.86 to 1.31)

Results obtained from Cox regression models. * adjusted for age, sex, baseline type 2 diabetes, baseline hypertension, baseline body mass index (4 categories) and smoking status (3 categories) and stratified by recruitment center. Note: interactions with sex, age or baseline type 2 diabetes were not statistically significant.

**Table 4:** Subgroup Hazard ratios (95% CI) for the risk of cataract, according to the intervention group.

	Control Diet	Mediterranean Diet with EVOO	Mediterranean Diet with Nuts
	(*n* = 1890)	(*n* = 1998)	(*n* = 1914)
Age			
Participants aged < 60 years			
Cases/Person-years	5/1375	14/1420	6/1559
Multivariable adjusted rate ratio *	1 (ref.)	2.53 (0.88–7.24)	1.21 (0.35–4.20)
60 ≤ Participants aged < 70 years			
Cases/Person-years	85/6055	103/6806	94/6430
Multivariable adjusted rate ratio *	1 (ref.)	1.05 (0.78–1.40)	1.07 (0.80–1.44)
Participants aged ≥ 70 years			
Cases/Person-years	89/3204	89/3502	74/2730
Multivariable adjusted rate ratio *	1 (ref.)	0.90 (0.67–1.22)	1.01 (0.74–1.38)
Sex			
Men			
Cases/Person-years	59/4451	86/5137	67/5300
Multivariable adjusted rate ratio **	1 (ref.)	1.22 (0.87–1.70)	1.00 (0.70–1.43)
Women			
Cases/Person-years	120/6183	120/6591	107/5419
Multivariable adjusted rate ratio **	1 (ref.)	0.92 (0.71–1.19)	1.12 (0.86–1.45)
Body mass index			
<30 kg/m^2^			
Cases/Person-years	81/5412	111/6197	93/5932
Multivariable adjusted rate ratio ***	1 (ref.)	1.23 (0.92–1.64)	1.14 (0.84–1.54)
≥30 kg/m^2^			
Cases/Person-years	98/5222	95/5530	81/4786
Multivariable adjusted rate ratio ***	1 (ref.)	0.86 (0.65–1.15)	0.97 (0.72–1.31)

Results obtained from Cox regression models. * adjusted for age, sex, baseline type 2 diabetes, baseline hypertension, baseline body mass index (4 categories) and smoking status (3 categories) and stratified by recruitment center. ** adjusted for age, baseline type 2 diabetes, baseline hypertension, baseline body mass index (4 categories) and smoking status (3 categories) and stratified by recruitment center. *** adjusted for age, sex, baseline type 2 diabetes, baseline hypertension, and smoking status (3 categories) and stratified by recruitment center.

## Abbreviations

MedDiet	Mediterranean Diet
EVOO	Extra-virgin Olive Oil
CVD	Cardiovascular Disease
MUFA	Mono-unsaturated fatty acids
PUFA	Poly-unsaturated fatty acids
TAC	Total anti-oxidant capacity
